# Coronary and cerebral thrombosis in a young patient after mild COVID-19 illness: a case report

**DOI:** 10.1093/ehjcr/ytaa270

**Published:** 2020-10-01

**Authors:** Lauren S Ranard, David J Engel, Ajay J Kirtane, Amirali Masoumi

**Affiliations:** Department of Medicine, Division of Cardiology, Columbia University Irving Medical Center/NewYork-Presbyterian Hospital, New York, NY, USA; Department of Medicine, Division of Cardiology, Columbia University Irving Medical Center/NewYork-Presbyterian Hospital, New York, NY, USA; Department of Medicine, Division of Cardiology, Columbia University Irving Medical Center/NewYork-Presbyterian Hospital, New York, NY, USA; Clinical Trials Center, Cardiovascular Research Foundation, New York, NY, USA; Department of Medicine, Division of Cardiology, Columbia University Irving Medical Center/NewYork-Presbyterian Hospital, New York, NY, USA

**Keywords:** Acute coronary syndrome, Thrombo-embolism, COVID-19, Case report

## Abstract

**Background:**

COVID-19 has spread worldwide and has caused significant morbidity and mortality. Myocardial injury and thrombo-embolism are known complications for those with severe forms of disease. The incidence and risk factors for these complications for those patients who are asymptomatic or with mild forms of COVID-19 is unknown.

**Case summary:**

In this report we describe the case of a 35-year-old man with no past cardiac history who presented with chest pain and a high-sensitivity troponin level of 386 ng/L in the context of an unspecified mild viral illness 1 month previously. Diagnostic evaluation revealed a new cardiomyopathy, left ventricular thrombus, and mid right coronary artery thrombosis. The coronary thrombosis was treated with thrombectomy. SARS-CoV-2 antibodies returned positive. He initially did well post-procedure; however, prior to discharge, he developed a second arterial thrombo-embolism event, a middle cerebral artery stroke. He was treated with thrombectomy and remains hospitalized.

**Discussion:**

Recognition that mild COVID-19 can be complicated by subsequent cardiac injury and/or coagulopathy is important. As more people recover from this viral illness and return to normal activity levels, discussion among cardiac experts has begun regarding screening for occult myocardial injury in those who plan to resume competitive athletic activity. This case highlights the need for investigation regarding (i) the duration of thrombophilia after recovery from illness; (ii) the population that should receive thromboprophylaxis; and (iii) the duration of thromboprophylaxis therapy for COVID-19.

**Learning points**

- A high index of suspicion should be maintained for thrombo-embolic complications of COVID-19, even in potentially mild cases of the disease.
- The role of chemoprophylaxis for COVID-19 is not currently known, but cases such as this one indicate that there might be a role.
- Even after anticoagulation for an initial thrombotic event, careful monitoring for potential subsequent thrombo-embolic events is required.

## Introduction

Coronarvirus disease-2019 (COVID-19), caused by severe acute respiratory syndrome coronavirus 2 (SARS-CoV-2), has resulted in significant morbidity and mortality.^1^ Cardiovascular complications, specifically myocardial injury, heart failure, and thrombo-embolic complications, have been well described in patients with severe manifestations of COVID-19.^2^ In the hospitalized COVID-19 population, the estimated incidence of developing at least one thrombo-embolism event is described to be between 8% and 17%, and the specific incidence of arterial thrombosis is estimated to be 3.5–3.7%.^3^^,^^4^ Most reported arterial thrombosis events involve peripheral arterial embolism and cerebrovascular infarction.^5^ Multivessel *in situ* coronary thrombosis in the setting of severe COVID-19 illness requiring hospitalization has been described in one case report.^6^ To our knowledge, there are no reports describing myocardial injury and thrombosis in patients with mild forms of COVID-19.

## Timeline

**Table:** 

29 days prior to admission	First presentation to medical care with symptoms of dyspnoea, fever, cough, and non-specific chest discomfort.
20 days prior to admission	Resolution of the above symptoms.
Day 1	Presentation to the Emergency Department with acute coronary syndrome and NSTEMI.
Day 1	TTE demonstrates reduced LV function and a mobile LV apical thrombus.
Day 1	Given ongoing pain, patient taken for coronary angiography; coronary angiography demonstrates mid-RCA thrombotic occlusion. The patient was treated with thrombectomy and admitted to the cardiac ICU.
Day 2	SARS-CoV-2 antibodies return positive.
Day 2	Patient is transferred to general cardiology floor.
Day 8	The patient was found in his room with acute left-sided hemineglect; CTA head and neck demonstrates right MCA thrombus.
Day 8	Emergent MCA thrombectomy performed.
Day 9	TTE post-event demonstrates ongoing LV dysfunction with LV thrombus smaller in size and less mobile.
Day 14	Remains hospitalized with residual neurological deficits requiring acute rehabilitation placement.

## Case presentation

A 35-year-old man with mild intermittent asthma, class I obesity, and no additional personal/familial cardiovascular risk factors presented to the emergency department (ED) complaining of 1 day of intermittent chest discomfort with associated left-sided jaw pain and nausea. The symptoms began while at rest on the morning prior to admission, subsided through the day, and returned with increased severity in the evening, leading to ED presentation.

The patient was in a usual state of health until 1 month previously; at that time, he developed symptoms of cough, non-specific chest pain, dyspnoea, and intermittent fevers (*T*_max_ 38.78°C). His albuterol inhaler provided only mild relief of symptoms. A chest radiograph (CXR) performed at an urgent care centre was reportedly unremarkable. He was presumed to have COVID-19 as well as an asthma exacerbation; however, he did not receive COVID-19 testing. He was prescribed a 5-day course of azithromycin and a combination inhaler (ipratropium bromide and albuterol). His symptoms resolved within 1 week, and he felt well until the day prior to index presentation.

In the ED, temperature was 37.6°C, heart rate 66 b.p.m., respiratory rate 18 breaths/min, and oxygen saturation was 99% on ambient air. Blood pressure was 123/81 mmHg (left arm) and 126/80 mmHg (right arm). The physical exam was unremarkable.

**Figure 1:**
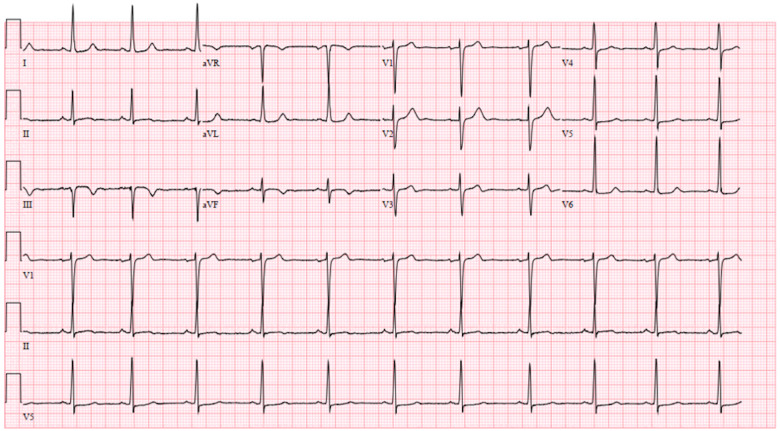
Admission electrocardiogram. Electrocardiogram showing sinus rhythm with inferior T-wave inversion and non-specific lateral ST changes.

The initial work-up in the ED consisted of a 12-lead electrocardiogram (ECG), laboratory testing (*Table 1*), and CXR. The ECG demonstrated sinus rhythm with inferior T-wave inversions, and non-specific lateral ST depression (*Figure 1*). Initial laboratory tests were remarkable for a high-sensitivity troponin T (hs-cTnT) of 386 ng/L (normal: ≤22 ng/L). The CXR showed a normal cardiac silhouette and clear lung fields. Given the elevated hs-cTnT, the patient received 325 mg aspirin and 80 mg enoxaparin.

**Table 1:** Laboratory values

Variable	Reference range	On admission
White blood cells	3120–8440/μL	7810
Haemoglobin	12.6–17.0 g/dL	15.4
Platelets	156 000–325 000/μL	215 000
Sodium	137–145 mmol/L	139
Potassium	3.5–5.1 mmol/L	4.7
Carbon dioxide	19–27 mmol/L	21
Blood urea nitrogen	7–26 mg/dL	13
Creatinine	0.70–1.30 mg/dL	1.12
Glucose level	75–100 mg/dL	140
Troponin T, high sensitivity	≤22 ng/L	386
Prothrombin time	11.9–14.4 s	13.2
International normalized ratio	0.9–1.1	1.0
Activated partial thromboplastin time	23.9–34.7 s	28.5
Total cholesterol	<200 mg/dL	220
Triglyceride	≤149 mg/dL	126
HDL	40–60 mg/dL	48
LDL		147 mg/dL
N-terminal probrain natriuretic peptide	0–93 pg/mL	315.8
Thyroid-stimulating hormone	0.41–4.81 mIU/L	4.02
Haemoglobin A1c	<5.7%	6.0
Erythrocyte sedimentation rate	0–15 mm/h	26
Ferritin	30–400 ng/mL	264.8
C-reactive protein, high sensitivity	0–10 mg/L	5.96
D-Dimer	≤0.80 μg/mL	0.50

**Figure 2:**
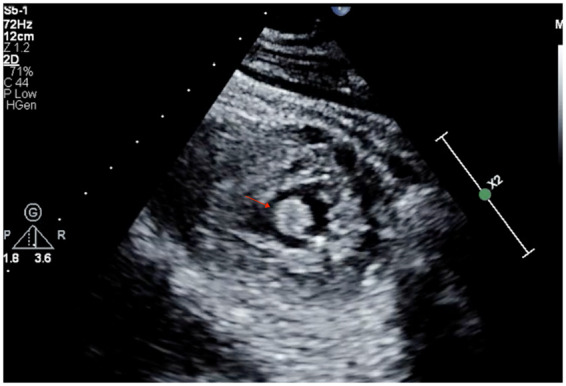
Transthoracic echocardiogram. Parasternal short-axis view demonstrating apical left ventricle thrombus (arrow).

Subsequent hs-cTnT increased to 502 ng/L, and then to 768 ng/L. A nasopharyngeal swab for SARS-CoV-2 RNA was negative. Transthoracic echocardiogram (TTE) demonstrated a mildly dilated left ventricle [left ventricular (LV) end-diastolic diameter 5.9 cm] and global hypokinesis [ejection fraction (EF) 20–25%] but akinetic inferior and inferolateral walls. A 2.0 cm × 0.7 cm mobile thrombus was visualized in the LV apex (*Figure 2*; Supplementary material online, *Video S1*). The patient underwent urgent left heart catheterization.

Coronary angiography revealed a mid right coronary artery (RCA) thrombus causing 100% occlusion with TIMI 0 flow (Supplementary material online, *Video S2*). The left coronary anatomy had no coronary disease and demonstrated no retrograde filling of the RCA. After discussion regarding pros and cons of intervention in this setting, reperfusion was elected given the ongoing symptoms. Intravenous cangrelor bolus was administered (2550 μg). Mechanical aspiration thrombectomy and subsequent mechanical-power aspiration thrombectomy were performed utilizing the Indigo System with CAT RX Coronary Aspiration Catheter (Penumbra). Post-thrombectomy, a large amount of residual thrombus burden remained, causing 70% residual stenosis and TIMI 2 flow (Supplementary material online, *Video S3*). At this time, the cangrelor bolus was complete and the patient was administered a loading dose of 180 mg ticagrelor, and started on a tirofiban infusion (loading dose of 2125 μg followed by 12.75 μg/min for 18 h).

**Figure 3:**
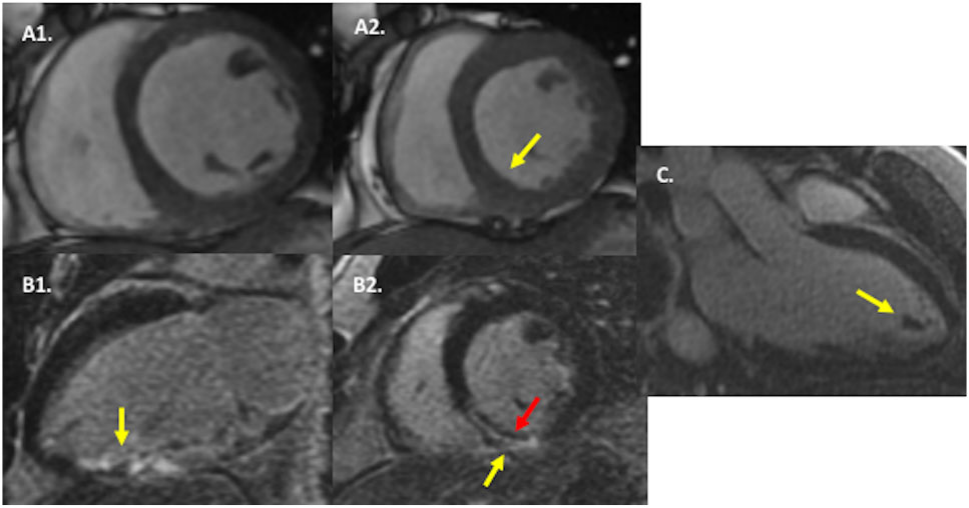
Cardiac magnetic resonance (CMR) imaging findings. In (*A*), cine CMR in mid-ventricular short-axis at end-diastole (A1) and end-systole (A2) demonstrates severe hypokinesis of the inferior and inferoseptal walls (yellow arrow) as well as mild global hypokinesis. In (*B*), late gadolinium enhancement CMR in two-chamber long-axis (B1) and mid-ventricular short-axis (B2) orientations reveals near transmural hyperenhancement in the basal–mid inferior and inferoseptal walls (yellow arrow), with a dark central zone (red arrow), consistent with transmural infarction with microvascular obstruction. In (*C*), late gadolinium enhancement CMR in three-chamber long-axis orientation shows a non-enhancing intracardiac mass in the left ventricular apex, consistent with thrombus.

On admission to the cardiac care unit, he was haemodynamically stable. Subsequently, SARS-CoV-2 antibody testing (Elecsys anti-SARS-CoV-2 test, electrochemiluminescence immunoassay) returned positive. Cardiac magnetic resonance imaging with late gadolinium enhancement tissue characterization demonstrated LVEF of 39%, and near transmural myocardial infarction of the basal–mid inferoseptum and inferior segments with a subendocardial non-enhancing central area suggestive of microvascular obstruction (*Figure 3*). There was no evidence of remote myocardial inflammation on T2-weighted or late gadolinium enhancement imaging.

The patient remained chest pain free post-coronary intervention, completed the 18-h tirofiban infusion, and was moved to the cardiology wards. Serial ECGs performed throughout the admission demonstrated non-dynamic ST and T wave changes, unchanged from that shown in *Figure 1*. On hospital Day 6, he developed left-sided hemineglect, confusion, and slurred speech. His antiplatelet/anticoagulation regimen at this time was aspirin 81 mg daily, clopidogrel 75 mg daily, unfractionated heparin infusion [activated partial thromboplastin time (aPTT) 84.0 s; normal 23.9–34.7 s] and warfarin [international normalized ratio (INR) 1.2; normal 0.9–1.1). Computed tomography head angiography demonstrated a right middle cerebral artery (MCA; M1 branch) thrombus (Supplementary material online, *Image S1*). No carotid disease was visualized. The patient was taken for emergent thrombectomy (Supplementary material online, *Image S2*). Follow-up TTE demonstrated LV apical thrombus, however smaller in size. Post-thrombectomy, he remains with mild left-sided weakness. The patient will be discharged to a rehabilitation facility with planned repeat echocardiogram and thrombophilia testing at 1-month follow-up.

## Discussion

This is a 35-year-old man who presented with newly reduced EF, LV thrombus, and acute coronary thrombosis in the setting of a prior viral illness during the peak of the COVID-19 pandemic. His SARS-CoV-2 antibodies returned positive. suggesting that his recent viral illness was COVID-19.

Those with severe COVID-19 are likely to have a predisposition to thrombosis related to excessive inflammation, hypoxia, immobilization, and diffuse intravascular coagulation. Endotheliopathy appears to contribute to the pathophysiology of microcirculatory changes in SARS-CoV-2 infections as the receptor for viral adhesion is an angiotensin-converting enzyme 2 receptor present on endothelial cells.^7^ Viral replication can cause inflammatory cell infiltration and endothelial cell apoptosis, leading to microvascular prothrombotic effects and microcirculatory dysfunction and thrombo-embolism.^8^ However, the role of this process in those with mild illness or who have recently recovered from COVID-19 infection is unknown.

This patient’s reduced EF probably pre-dates this acute coronary syndrome (ACS) presentation and accounts for the formation of the large apical thrombus. The normal D-dimer additionally suggests chronicity to his presentation. Although there were no signs of inflammatory myocarditis on cardiac magnetic resonance imaging with non-elevated inflammatory biomarkers, an incomplete recovery of EF after myocarditis remains possible. An endomyocardial biopsy was not performed for further evaluation given the risks associated with performing such a procedure on therapeutic anticoagulation. Possible sequences of events leading up to this patient’s presentation include (i) embolization of LV thrombus to the RCA causing ACS; or (ii) an independent thrombotic phenomenon in both the left ventricle and RCA.

Recognition that COVID-19 associated with mild symptoms can be complicated by subsequent cardiac injury and/or coagulopathy is important. As more people recover from this viral illness and return to normal activity levels, discussion among cardiac experts has begun regarding screening for occult myocardial injury in those who plan to resume intense and competitive athletic activity. For example, the ACC Sports and Exercise Cardiology Council has put forward a return-to-play guideline algorithm for competitive athletes and highly active people who contracted COVID-19 with mild or severe disease that includes hs-cTnT, TTE, and ECG testing prior to returning to play.^9^ This patient’s presentation provides support for this conservative approach. Lastly, this case highlights the need for investigation regarding (i) the duration of thrombotic derangements after recovery from illness; (ii) the population that should receive thromboprophylaxis; and (iii) the duration of thromboprophylaxis therapy in COVID-19.

## Conclusion

Patients with mild forms of COVID-19 remain at risk for COVID-19-associated cardiac injury and coagulopathy. More studies are needed to identify those at risk for COVID-19-associated coagulopathy and occult myocardial injury.

## Lead author biography

**Figure:**
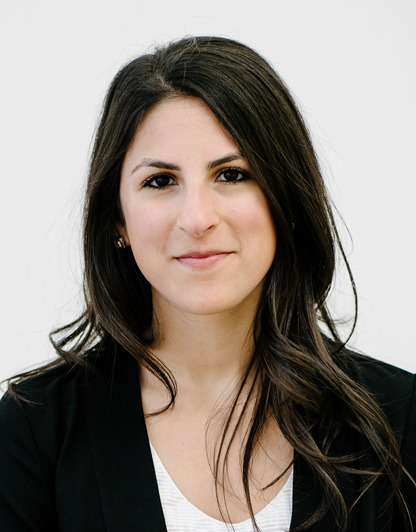


Lauren S. Ranard, MD, is a cardiology fellow at Columbia University Irving Medical Center/NewYork-Presbyterian Hospital. She completed her undergraduate training at Cornell University in Ithaca, New York, medical school at Jefferson Medical College in Philadelphia, Pennsylvania and residency in Internal Medicine at Duke University Medical Center in Durham, North Carolina. Her research interests include acute coronary syndrome and structural heart disease.

## Supplementary material

Supplementary material is available at *European Heart Journal – Case Reports* online.

## Supplementary Material

ytaa270_Supplementary_Data
