# Supplementary material for: Coronary and cerebral thrombosis in a young patient after mild COVID-19 illness: a case report
Source: Eur Heart J Case Rep. 2020 Oct 1;4(5):1–5. doi: 10.1093/ehjcr/ytaa270 (PMC7543370; doi:10.1093/ehjcr/ytaa270)
Supplement: ytaa270_Supplementary_Data [file ytaa270_supplementary_data.zip › ytaa270-suppl_data/Slide Set COVID Thrombosis v2.pptx]

## Slide 1
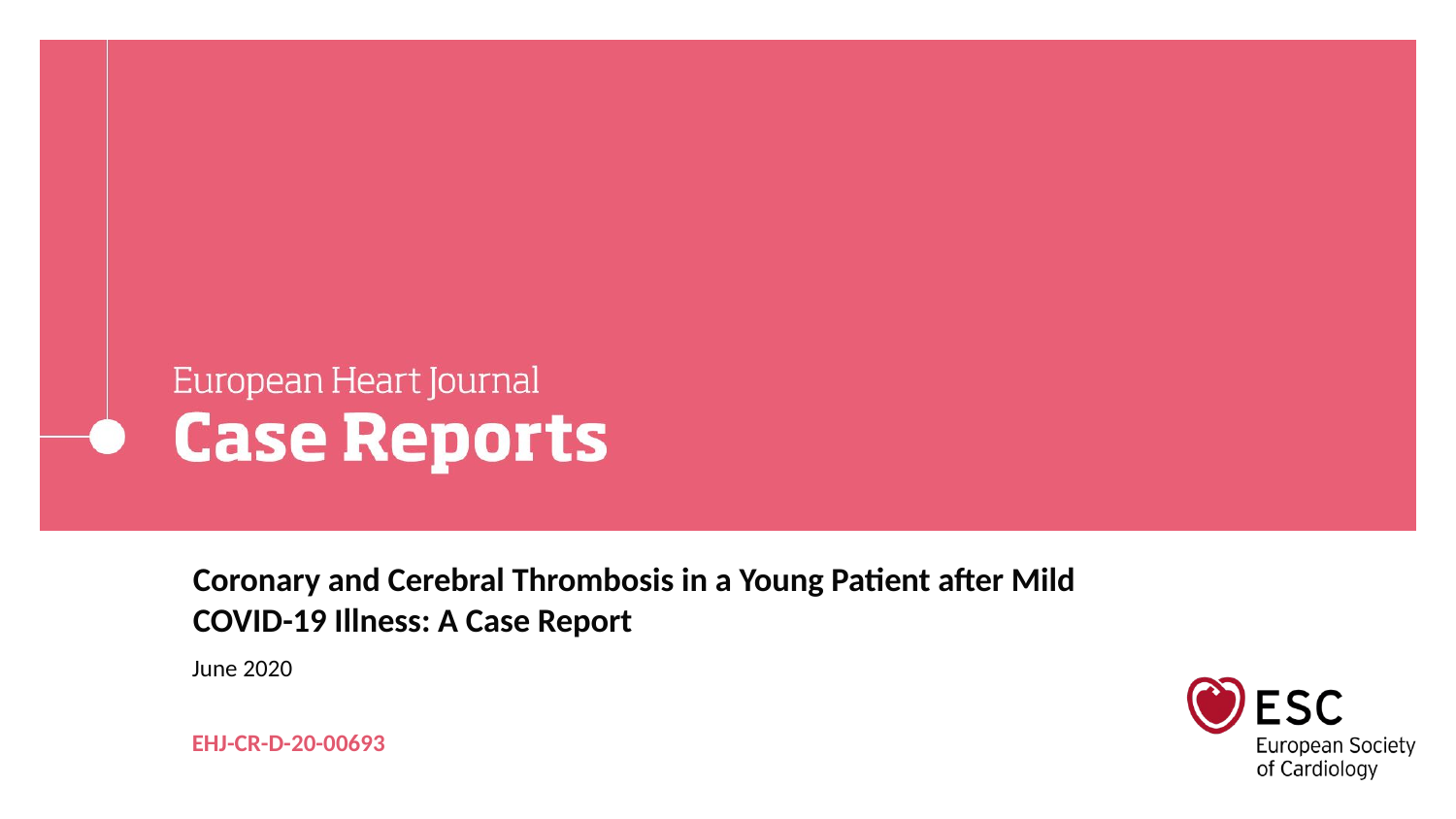

# Coronary and Cerebral Thrombosis in a Young Patient after Mild COVID-19 Illness: A Case Report
June 2020
EHJ-CR-D-20-00693

## Slide 2
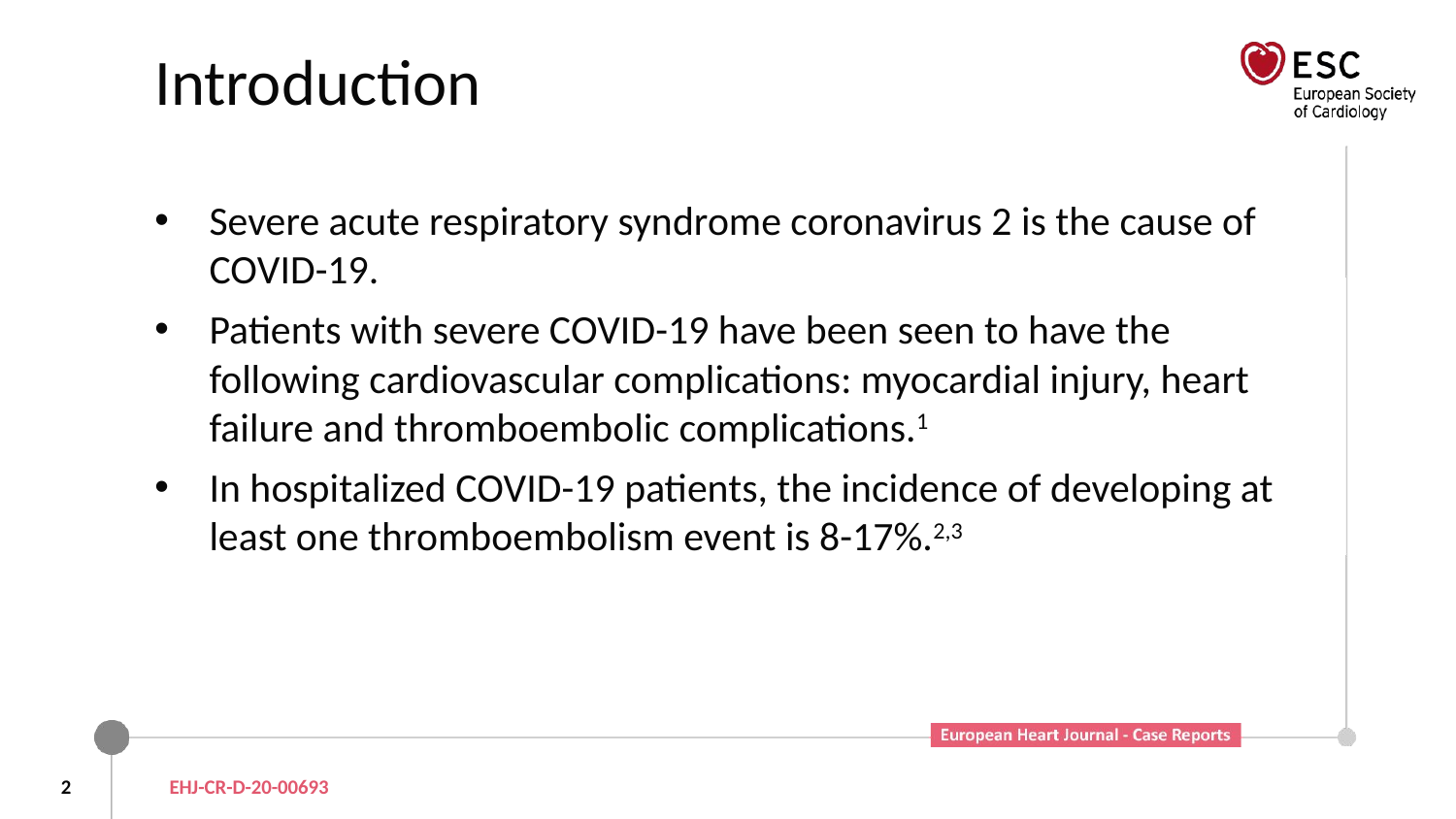

# Introduction
Severe acute respiratory syndrome coronavirus 2 is the cause of COVID-19.
Patients with severe COVID-19 have been seen to have the following cardiovascular complications: myocardial injury, heart failure and thromboembolic complications.1
In hospitalized COVID-19 patients, the incidence of developing at least one thromboembolism event is 8-17%.2,3
2
EHJ-CR-D-20-00693

## Slide 3
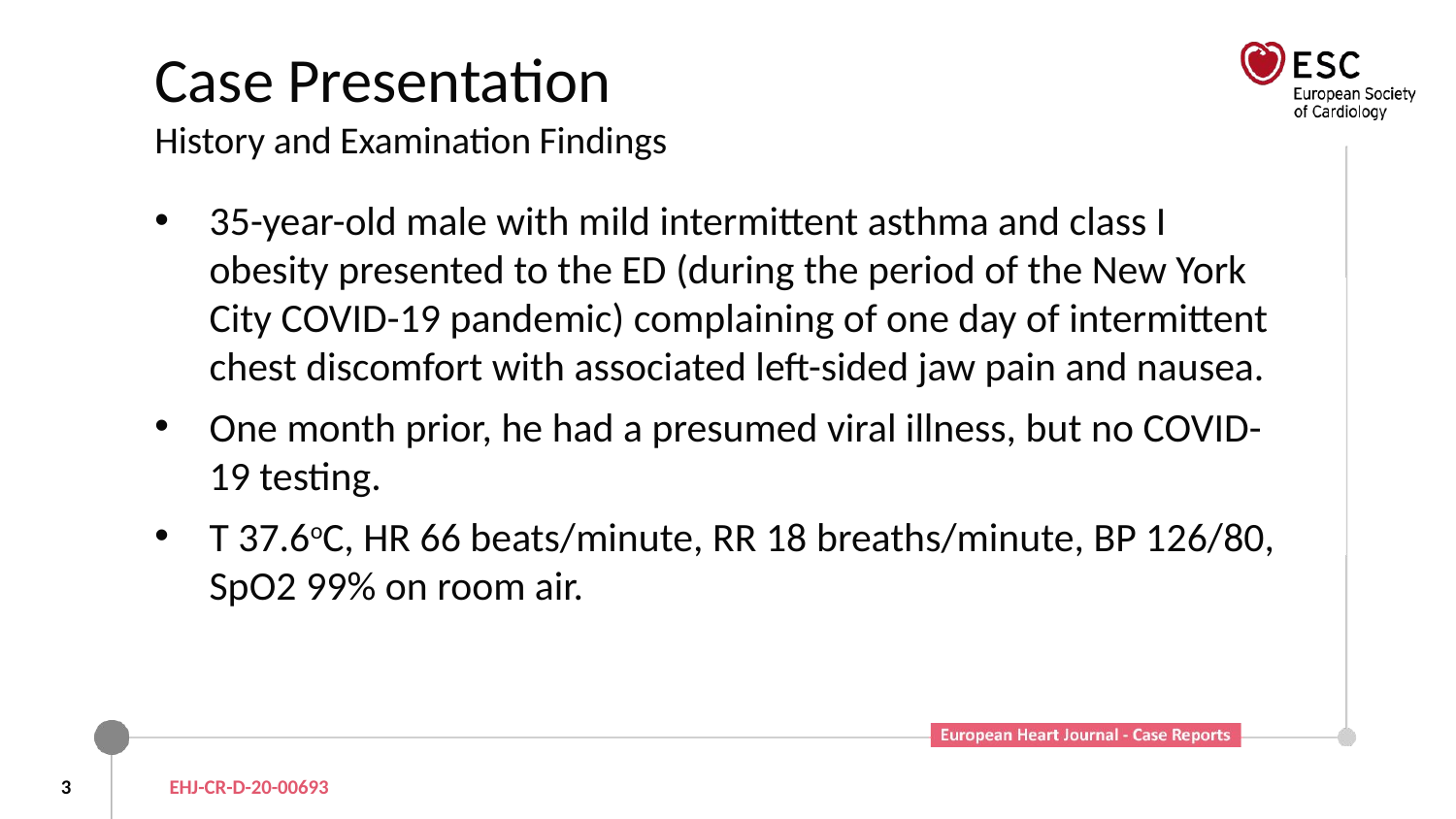

# Case Presentation History and Examination Findings
35-year-old male with mild intermittent asthma and class I obesity presented to the ED (during the period of the New York City COVID-19 pandemic) complaining of one day of intermittent chest discomfort with associated left-sided jaw pain and nausea.
One month prior, he had a presumed viral illness, but no COVID-19 testing.
T 37.6oC, HR 66 beats/minute, RR 18 breaths/minute, BP 126/80, SpO2 99% on room air.
3
EHJ-CR-D-20-00693

## Slide 4
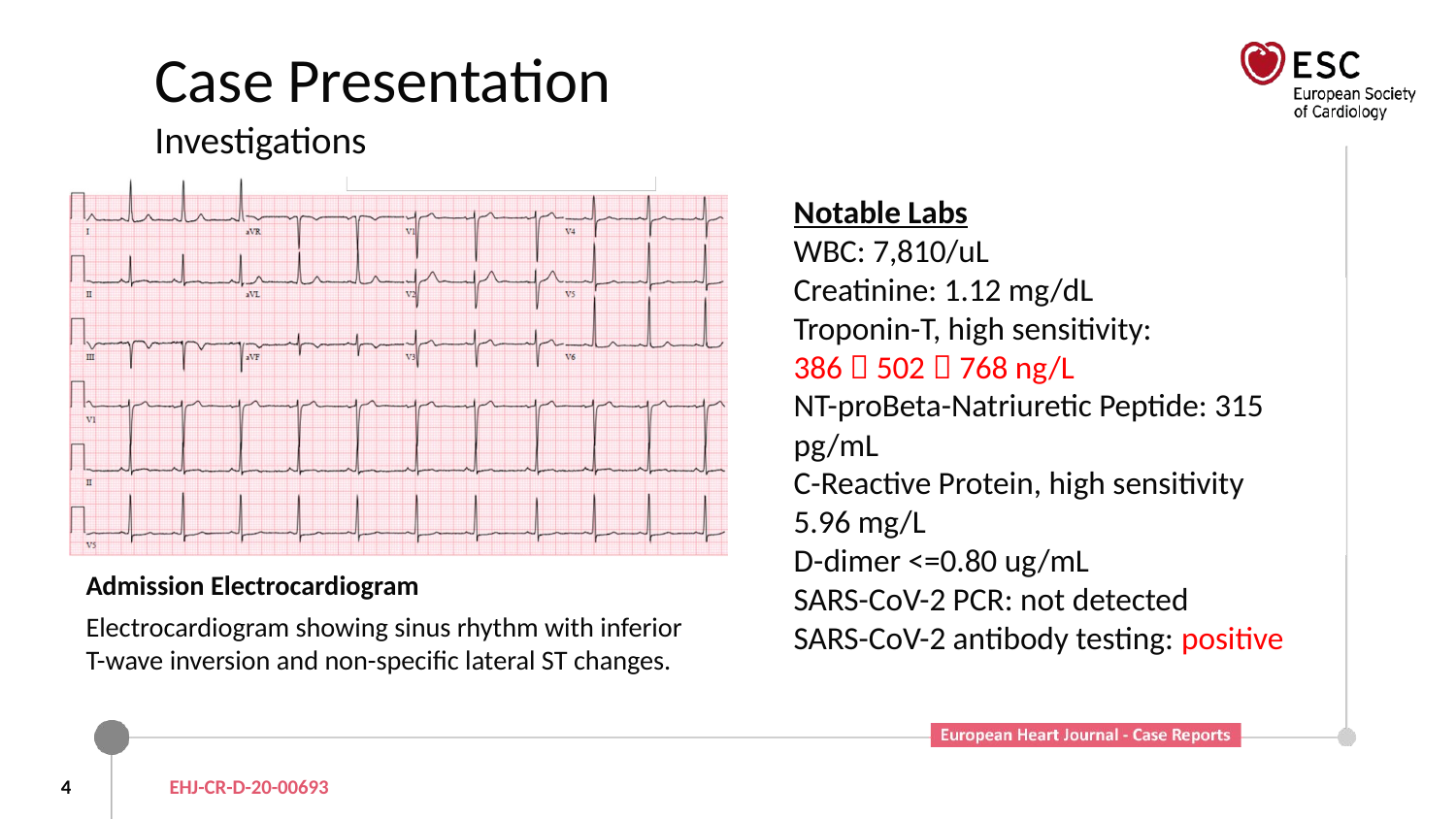

# Case Presentation Investigations
Notable Labs
WBC: 7,810/uL
Creatinine: 1.12 mg/dL
Troponin-T, high sensitivity:
386  502  768 ng/L
NT-proBeta-Natriuretic Peptide: 315 pg/mL
C-Reactive Protein, high sensitivity 5.96 mg/L
D-dimer <=0.80 ug/mL
SARS-CoV-2 PCR: not detected
SARS-CoV-2 antibody testing: positive
Admission Electrocardiogram
Electrocardiogram showing sinus rhythm with inferior T-wave inversion and non-specific lateral ST changes.
4
EHJ-CR-D-20-00693

## Slide 5
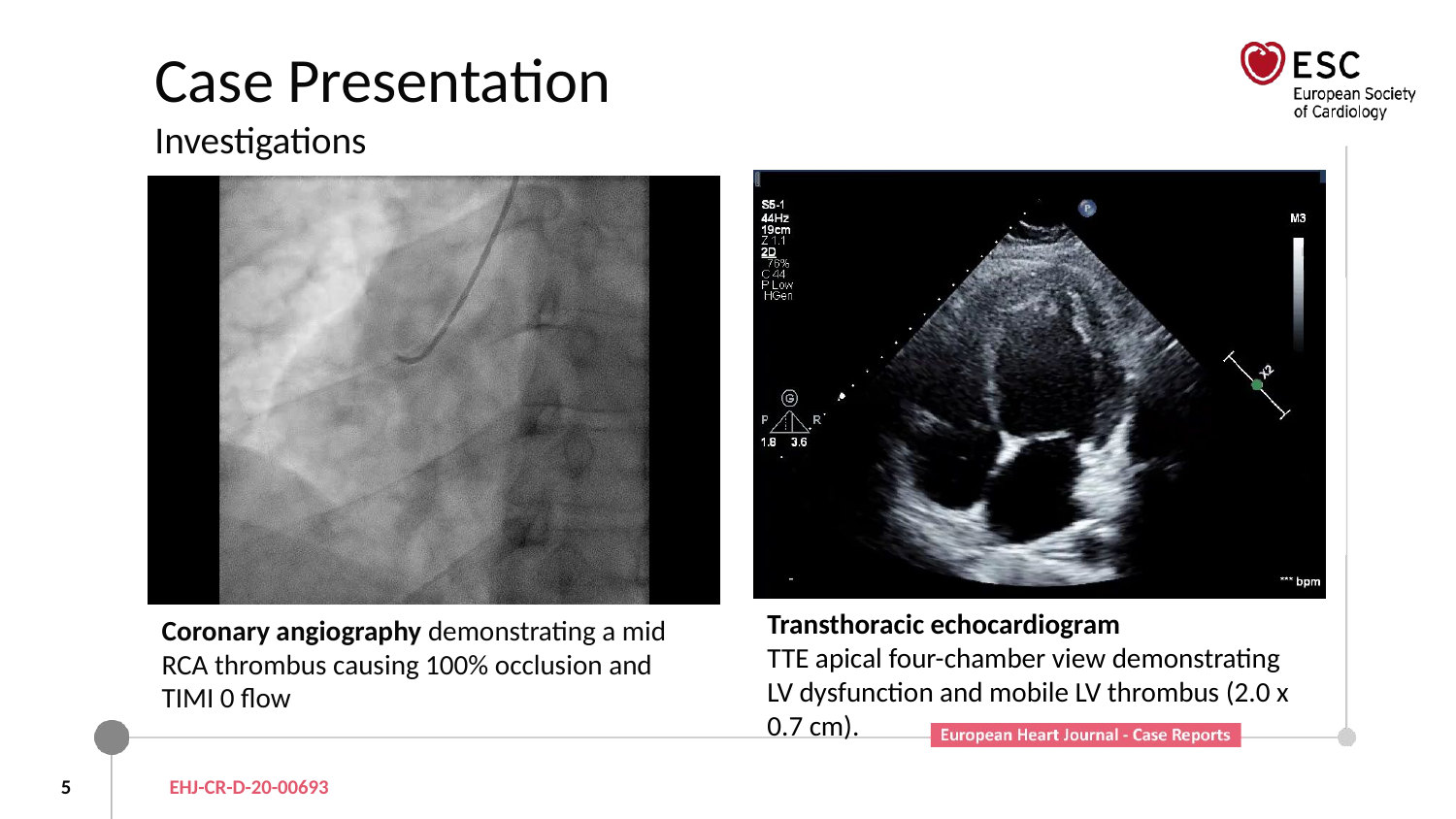

# Case Presentation Investigations
Transthoracic echocardiogram
TTE apical four-chamber view demonstrating LV dysfunction and mobile LV thrombus (2.0 x 0.7 cm).
Coronary angiography demonstrating a mid RCA thrombus causing 100% occlusion and TIMI 0 flow
5
EHJ-CR-D-20-00693

## Slide 6
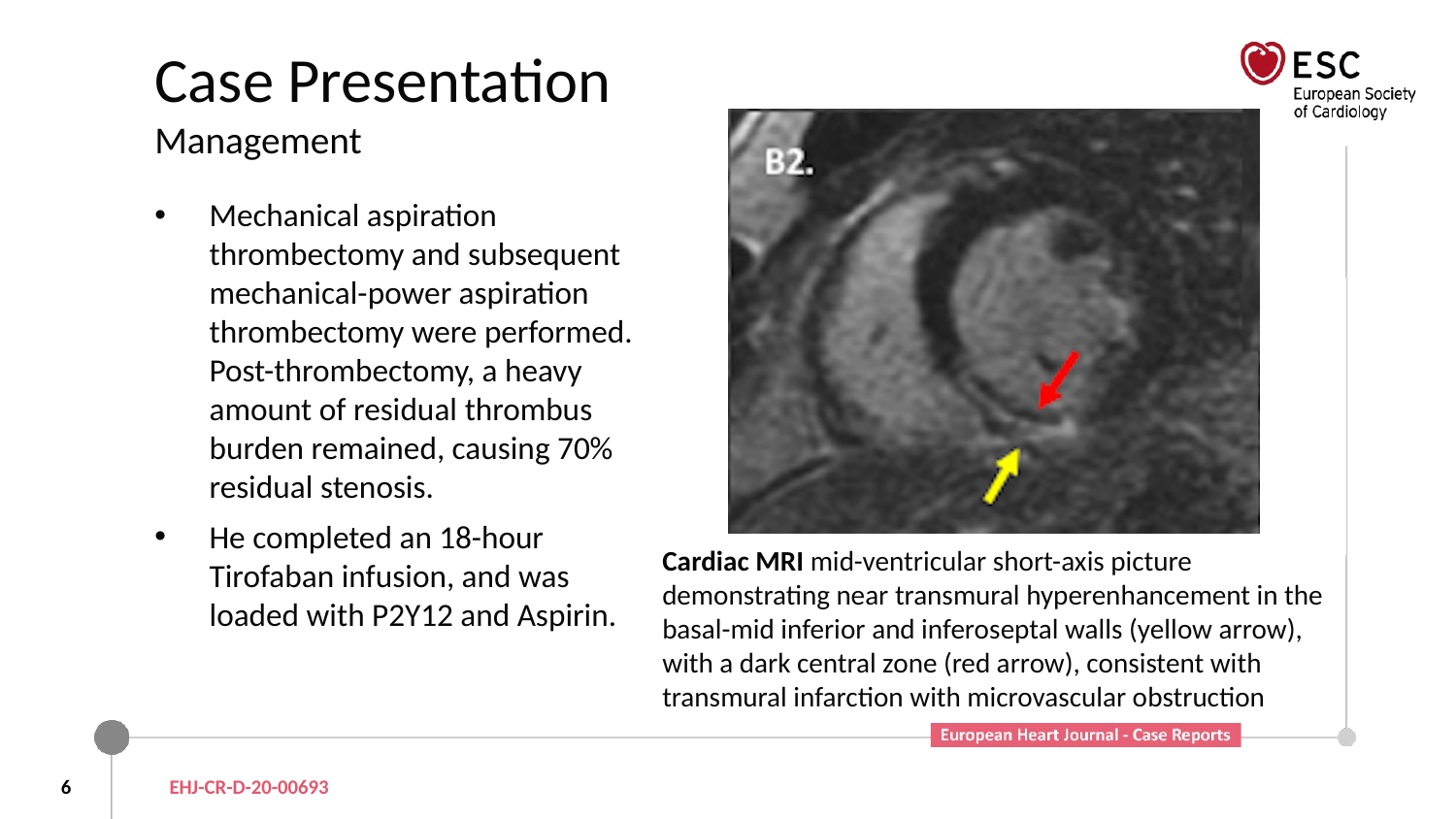

# Case Presentation Management
Mechanical aspiration thrombectomy and subsequent mechanical-power aspiration thrombectomy were performed. Post-thrombectomy, a heavy amount of residual thrombus burden remained, causing 70% residual stenosis.
He completed an 18-hour Tirofaban infusion, and was loaded with P2Y12 and Aspirin.
Cardiac MRI mid-ventricular short-axis picture demonstrating near transmural hyperenhancement in the basal-mid inferior and inferoseptal walls (yellow arrow), with a dark central zone (red arrow), consistent with transmural infarction with microvascular obstruction
6
EHJ-CR-D-20-00693

## Slide 7
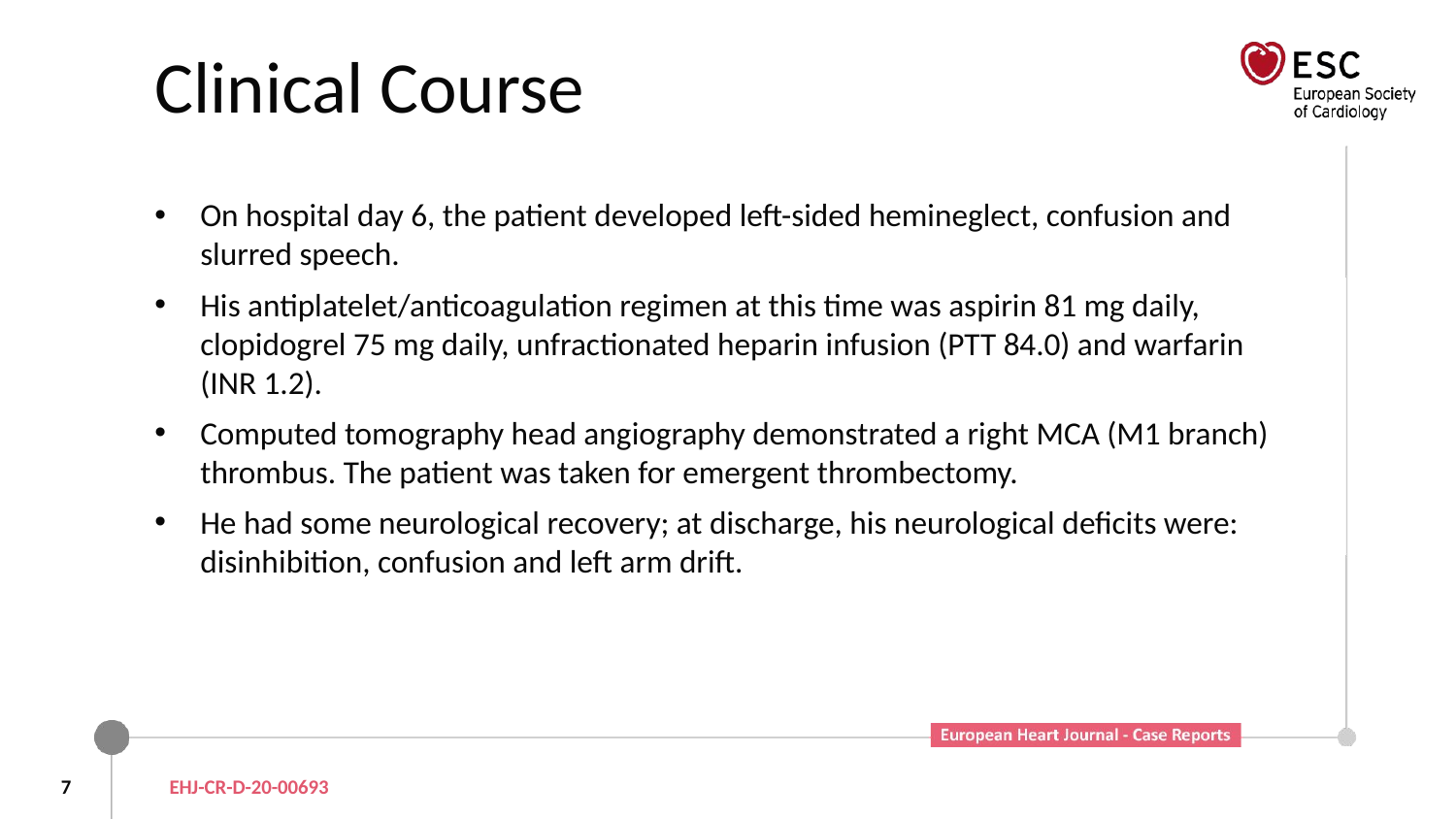

# Clinical Course
On hospital day 6, the patient developed left-sided hemineglect, confusion and slurred speech.
His antiplatelet/anticoagulation regimen at this time was aspirin 81 mg daily, clopidogrel 75 mg daily, unfractionated heparin infusion (PTT 84.0) and warfarin (INR 1.2).
Computed tomography head angiography demonstrated a right MCA (M1 branch) thrombus. The patient was taken for emergent thrombectomy.
He had some neurological recovery; at discharge, his neurological deficits were: disinhibition, confusion and left arm drift.
7
EHJ-CR-D-20-00693

## Slide 8
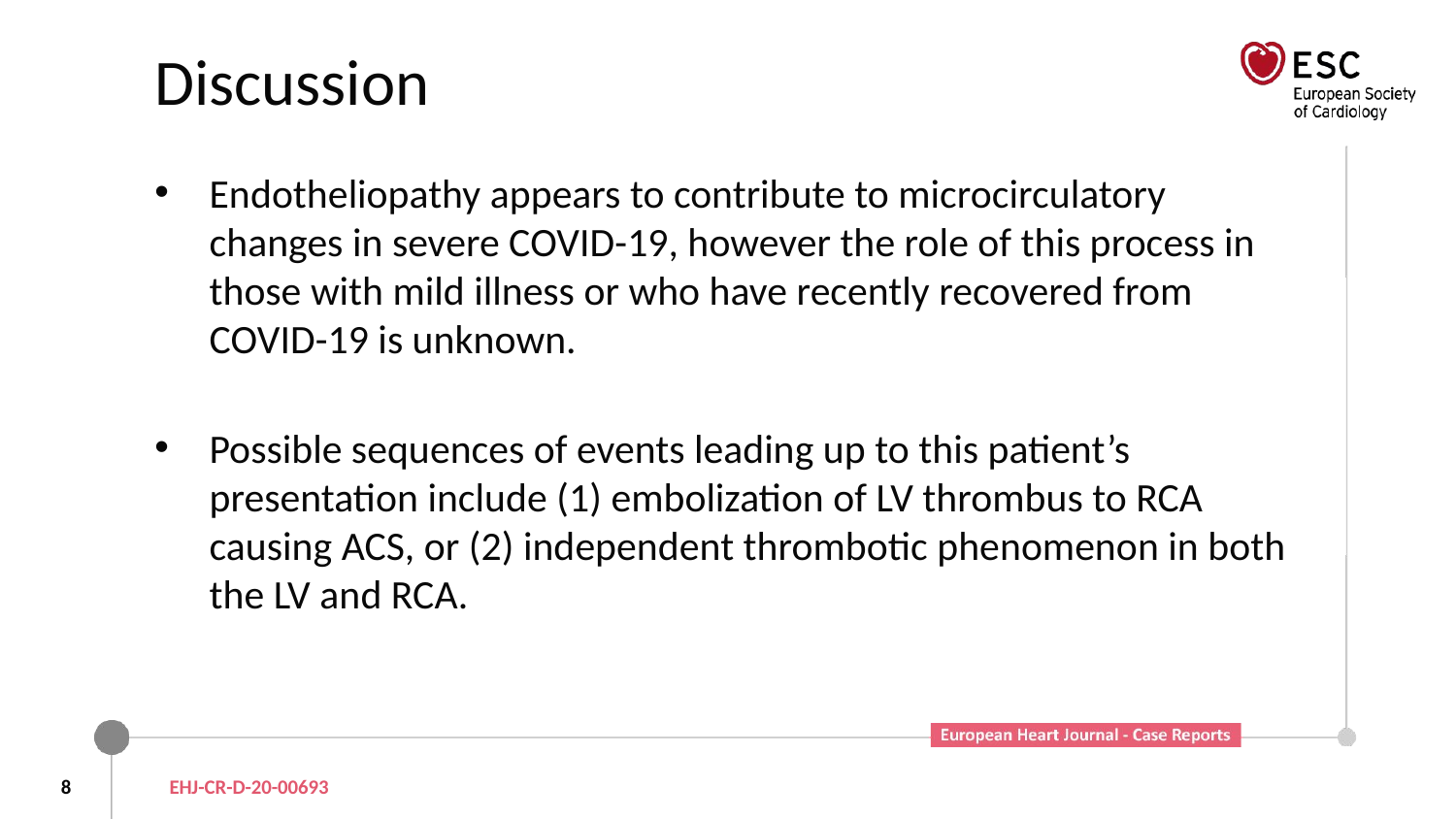

# Discussion
Endotheliopathy appears to contribute to microcirculatory changes in severe COVID-19, however the role of this process in those with mild illness or who have recently recovered from COVID-19 is unknown.
Possible sequences of events leading up to this patient’s presentation include (1) embolization of LV thrombus to RCA causing ACS, or (2) independent thrombotic phenomenon in both the LV and RCA.
8
EHJ-CR-D-20-00693

## Slide 9
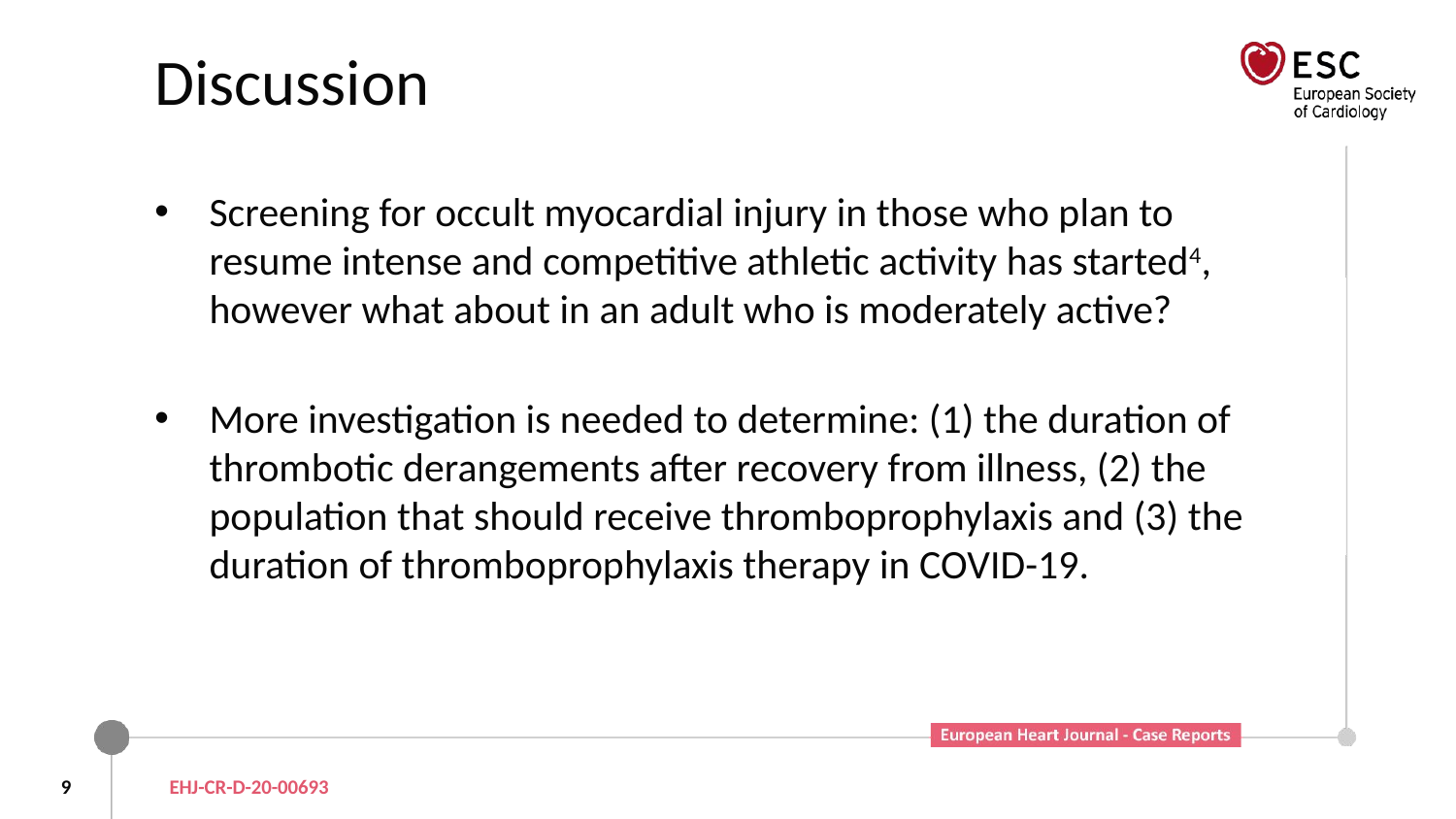

# Discussion
Screening for occult myocardial injury in those who plan to resume intense and competitive athletic activity has started4, however what about in an adult who is moderately active?
More investigation is needed to determine: (1) the duration of thrombotic derangements after recovery from illness, (2) the population that should receive thromboprophylaxis and (3) the duration of thromboprophylaxis therapy in COVID-19.
9
EHJ-CR-D-20-00693

## Slide 10
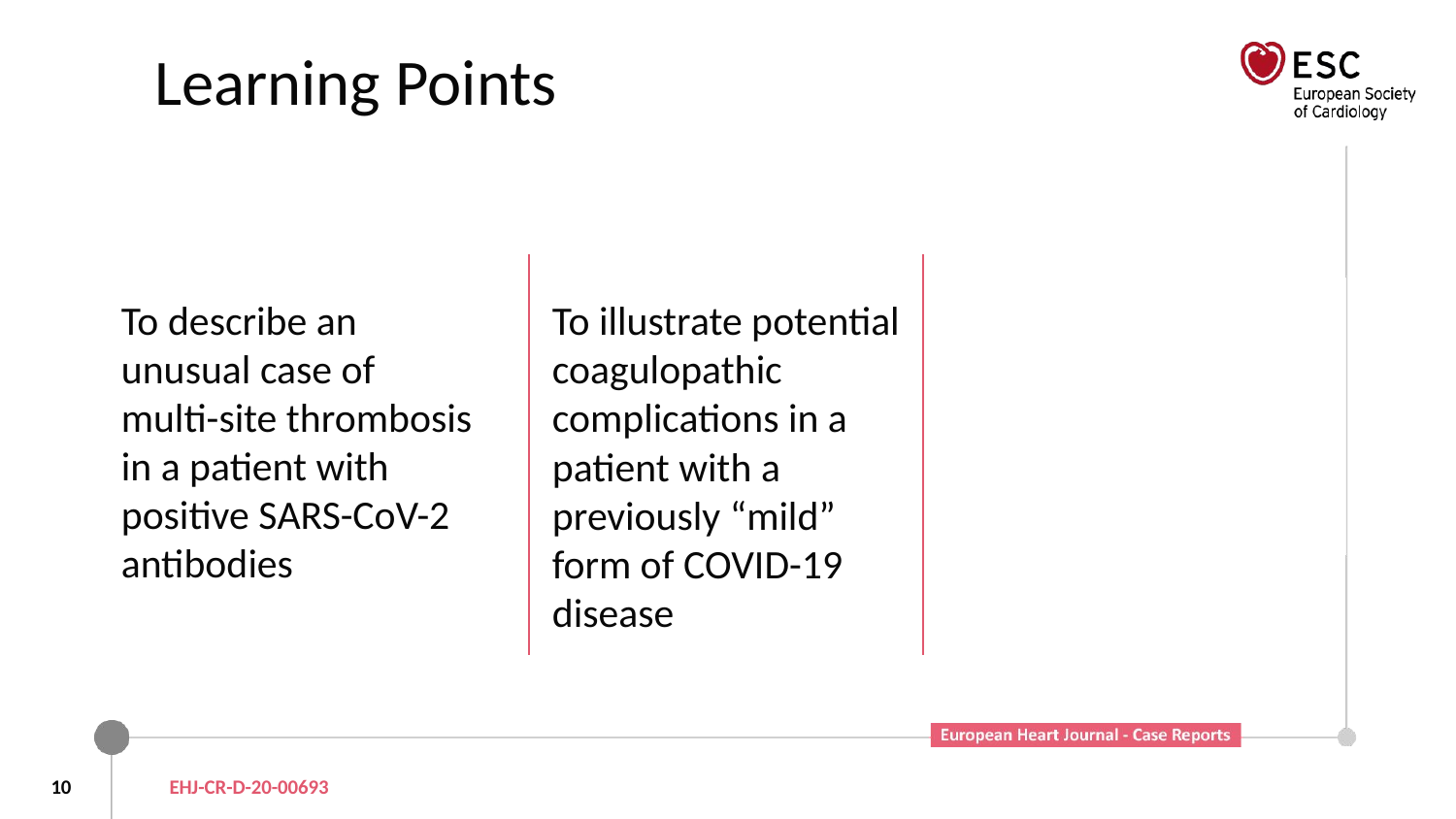

# Learning Points
To describe an unusual case of multi-site thrombosis in a patient with positive SARS-CoV-2 antibodies
To illustrate potential coagulopathic complications in a patient with a previously “mild” form of COVID-19 disease
10
EHJ-CR-D-20-00693

## Slide 11
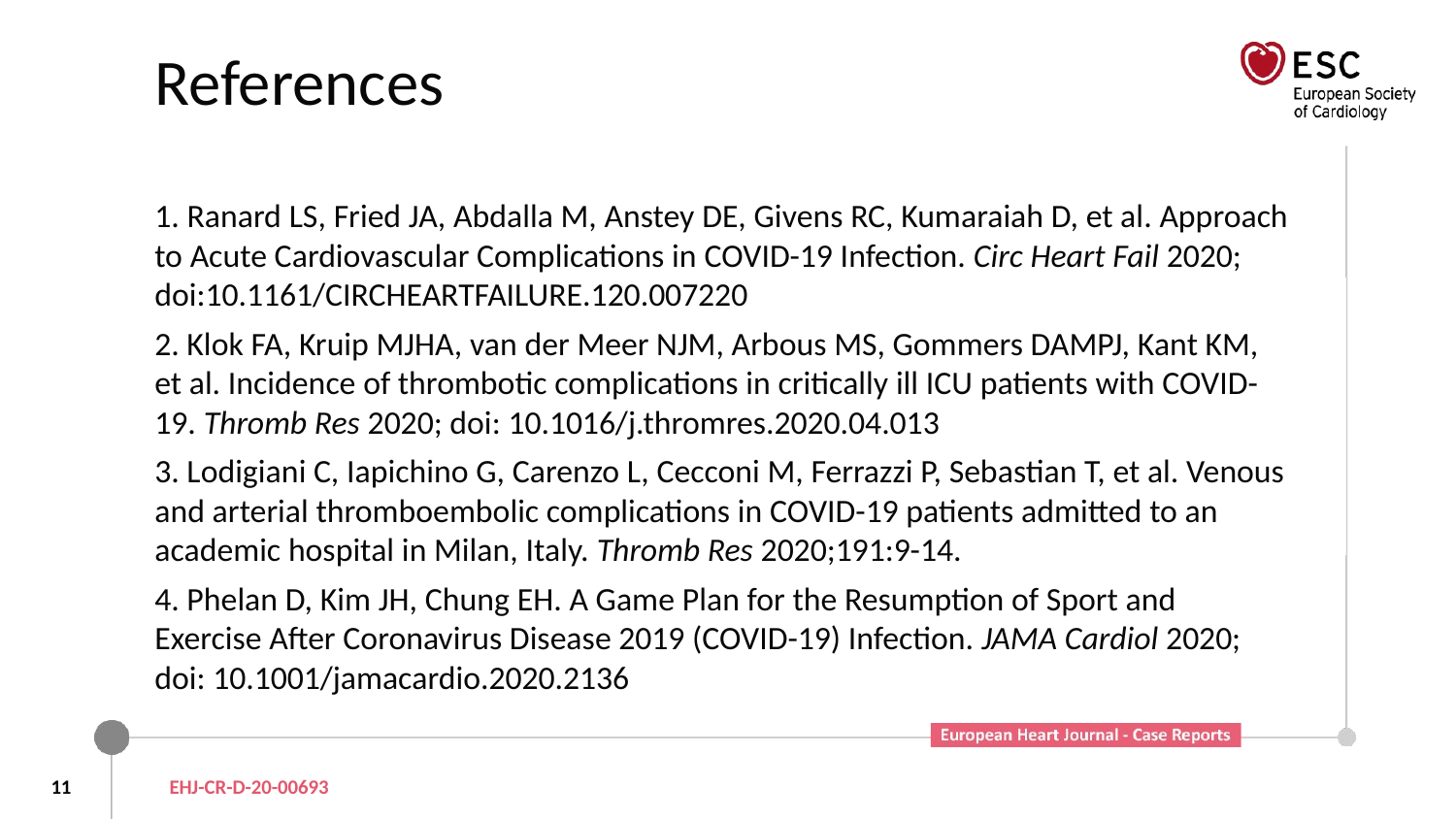

# References
1. Ranard LS, Fried JA, Abdalla M, Anstey DE, Givens RC, Kumaraiah D, et al. Approach to Acute Cardiovascular Complications in COVID-19 Infection. Circ Heart Fail 2020; doi:10.1161/CIRCHEARTFAILURE.120.007220
2. Klok FA, Kruip MJHA, van der Meer NJM, Arbous MS, Gommers DAMPJ, Kant KM, et al. Incidence of thrombotic complications in critically ill ICU patients with COVID-19. Thromb Res 2020; doi: 10.1016/j.thromres.2020.04.013
3. Lodigiani C, Iapichino G, Carenzo L, Cecconi M, Ferrazzi P, Sebastian T, et al. Venous and arterial thromboembolic complications in COVID-19 patients admitted to an academic hospital in Milan, Italy. Thromb Res 2020;191:9-14.
4. Phelan D, Kim JH, Chung EH. A Game Plan for the Resumption of Sport and Exercise After Coronavirus Disease 2019 (COVID-19) Infection. JAMA Cardiol 2020; doi: 10.1001/jamacardio.2020.2136
11
EHJ-CR-D-20-00693
