# Supplementary figures and images for: Coronary and cerebral thrombosis in a young patient after mild COVID-19 illness: a case report
Source: Eur Heart J Case Rep. 2020 Oct 1;4(5):1–5. doi: 10.1093/ehjcr/ytaa270 (PMC7543370; doi:10.1093/ehjcr/ytaa270)

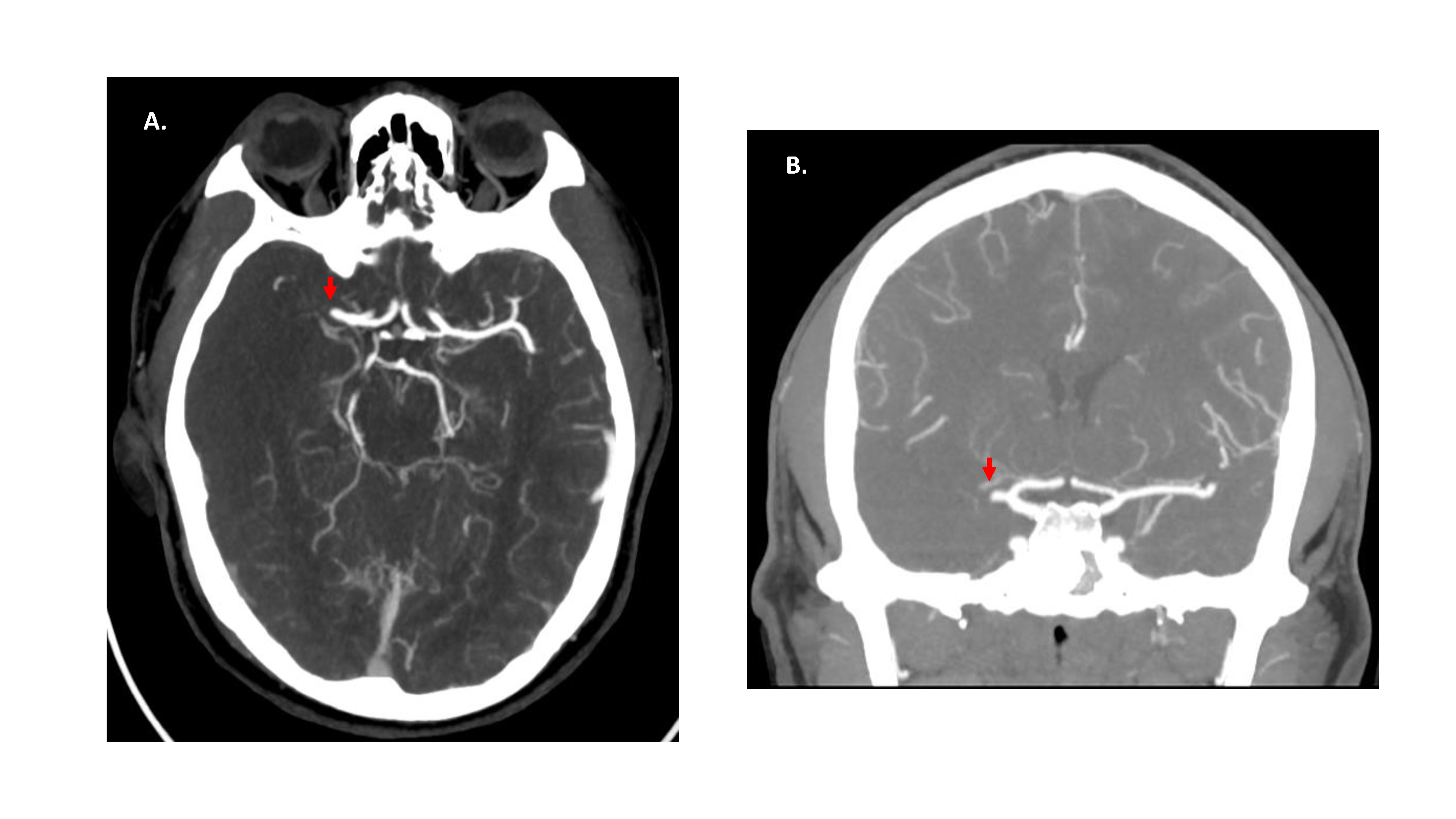

Supplement: ytaa270_Supplementary_Data [file ytaa270_supplementary_data.zip › ytaa270-suppl_data/Figure S1.png]

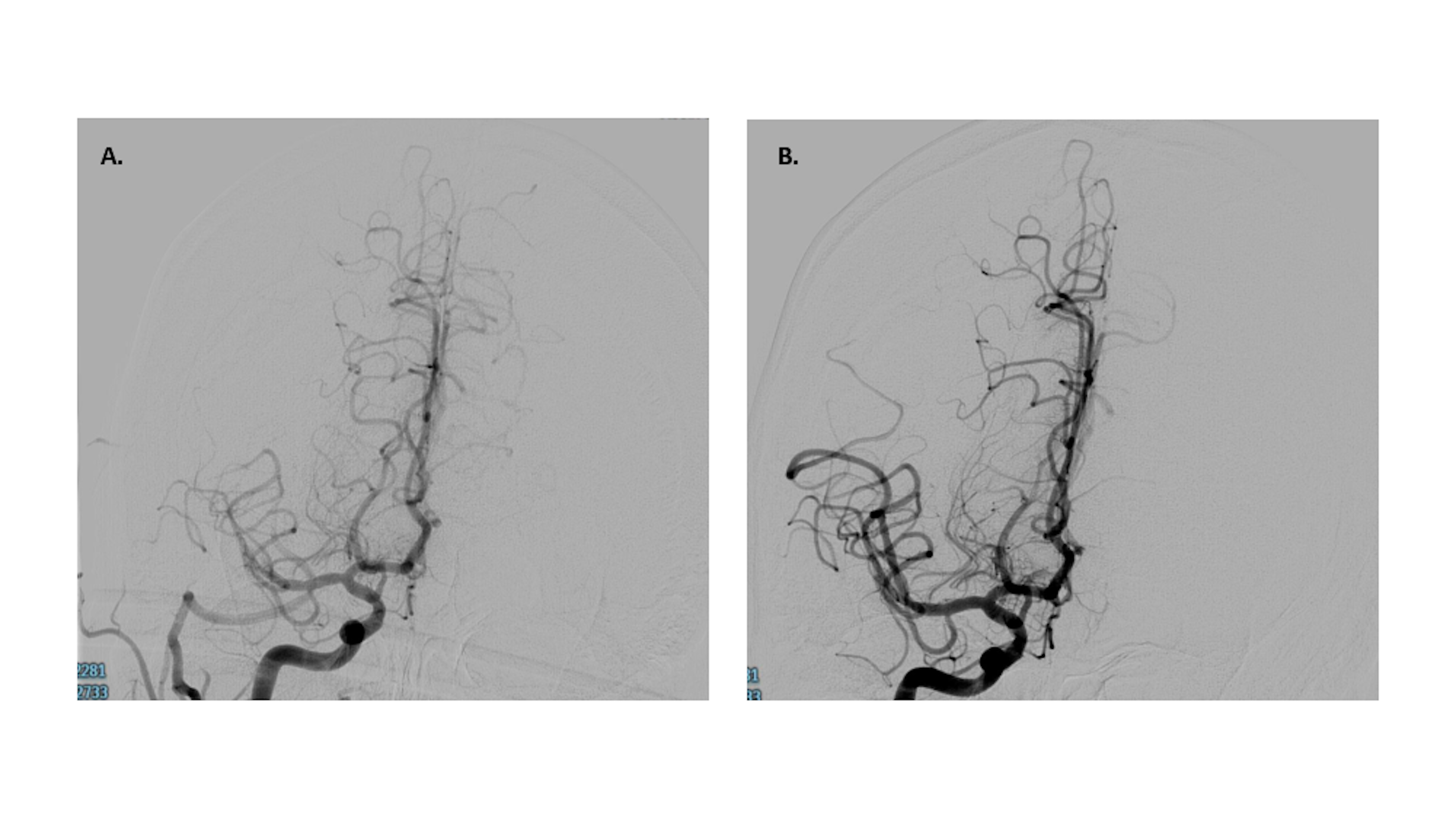

Supplement: ytaa270_Supplementary_Data [file ytaa270_supplementary_data.zip › ytaa270-suppl_data/Figure S2.png]
